# Feature extraction from MRI ADC images for brain tumor classification using machine learning techniques

**DOI:** 10.1186/s12938-022-01022-6

**Published:** 2022-08-01

**Authors:** Sahan M. Vijithananda, Mohan L. Jayatilake, Badra Hewavithana, Teresa Gonçalves, Luis M. Rato, Bimali S. Weerakoon, Tharindu D. Kalupahana, Anil D. Silva, Karuna D. Dissanayake

**Affiliations:** 1 Department of Radiology, Faculty of Medicine, University of Peradeniya, Peradeniya, Sri Lanka; 2 Department of Radiography and Radiotherapy, University of Peradeniya, Peradeniya, Sri Lanka; 3 Department of Informatics, University of Évora, Évora, Portugal; 4 Department of Computer Engineering, University of Sri Jayawardhanapura, Dehiwala-Mount Lavinia, Sri Lanka; 5 Epilepsy Unit, National Hospital of Sri Lanka, Colombo 10, Sri Lanka; 6 Department of Histopathology, National Hospital of Sri Lanka, Colombo 10, Sri Lanka

**Keywords:** Magnetic resonance imaging, Diffusion weighted imaging, Apparent diffusion coefficient, Brain tumor classification, ANOVA *f*-test feature selection, Machine learning, Random forest

## Abstract

**Background:**

Diffusion-weighted (DW) imaging is a well-recognized magnetic resonance imaging (MRI) technique that is being routinely used in brain examinations in modern clinical radiology practices. This study focuses on extracting demographic and texture features from MRI Apparent Diffusion Coefficient (ADC) images of human brain tumors, identifying the distribution patterns of each feature and applying Machine Learning (ML) techniques to differentiate malignant from benign brain tumors.

**Methods:**

This prospective study was carried out using 1599 labeled MRI brain ADC image slices, 995 malignant, 604 benign from 195 patients who were radiologically diagnosed and histopathologically confirmed as brain tumor patients. The demographics, mean pixel values, skewness, kurtosis, features of Grey Level Co-occurrence Matrix (GLCM), mean, variance, energy, entropy, contrast, homogeneity, correlation, prominence and shade, were extracted from MRI ADC images of each patient. At the feature selection phase, the validity of the extracted features were measured using ANOVA *f*-test. Then, these features were used as input to several Machine Learning classification algorithms and the respective models were assessed.

**Results:**

According to the results of ANOVA *f*-test feature selection process, two attributes: skewness (3.34) and GLCM homogeneity (3.45) scored the lowest ANOVA *f*-test scores. Therefore, both features were excluded in continuation of the experiment. From the different tested ML algorithms, the Random Forest classifier was chosen to build the final ML model, since it presented the highest accuracy. The final model was able to predict malignant and benign neoplasms with an 90.41% accuracy after the hyper parameter tuning process.

**Conclusions:**

This study concludes that the above mentioned features (except skewness and GLCM homogeneity) are informative to identify and differentiate malignant from benign brain tumors. Moreover, they enable the development of a high-performance ML model that has the ability to assist in the decision-making steps of brain tumor diagnosis process, prior to attempting invasive diagnostic procedures, such as brain biopsies.

## Background

Brain tumors are neoplastic tissue masses in which cells multiply and grow uncontrollably without being checked by the mechanisms that control normal cell division. It can occur at any age [1] and is one of the major diseases that affects the human central nervous system (CNS). According to a study done in United States, 29.9 per 100000 adults (20 years or older) are vulnerable to have a brain tumor at any stage of their life time [2]. Approximately one-third of these brain tumors are malignant and the others remain as benign tumors [2, 3].

Although the computed tomography (CT), positron emission tomography (PET) medical imaging techniques are frequently involved in brain tumor diagnosis process, MRI is considered the most effective tumor imaging method due to its superior contrast properties in current radiological practices [4]. However, the noise within medical images, including MRI ones, and non-systematic search of patterns by humans (radiologists) affects the accuracy of the diagnosis. Therefore, patients often need to go through invasive biopsy procedures to confirm, through histopathological analysis, the type (including its malignant or benign status) and the WHO grade of the tumour [5].

### Magnetic resonance imaging

Diffusion Weighted (DW) imaging is a form of magnetic resonance imaging (MRI) technique that is widely used in tumor identification and classification in modern clinical radiology practices [6, 7]. This technology is based on measurements of random Brownian motion of water molecules within a voxel of a biological tissue [8–10]. The technique allows to visualize the net direction of diffusion of water molecules or collective flow of water molecules in a live tissue. Hence, it has the ability to provide information on the microscopic behaviour of living biological tissues (such as the presence and permeability of membranes and the presence of macro-molecules and intracellular–extracellular water equilibrium) by measuring and imaging the transitional mobility of water molecules [11–14]. Due to the characteristic features of DW images, they are appreciated as an indispensable tool for investigating CNS diseases, such as brain neoplasms, brain and spinal cord injuries, degenerative brain diseases, etc.

The resistance for the diffusion of water molecules inside a tissue is quantitatively assessed by calculating the apparent diffusion coefficient (ADC) values [10]. To generate an ADC map, there should be at least two types of DW images differing from each other in terms of the diffusion sensitization level (*b* value). In most cases, it is common to utilize $b=0$ s/mm^2^ for the lower limit and images with *b* value in the range of 600 to $1000$ s/mm^2^ for the upper limit [15, 16]. However, there are evidences of using a *b* value greater than $1000$ s/mm^2^ as the upper limit of ADC image generation [17].

### Texture features

The generated ADC images reflect the magnitude of diffusion of water molecules within tissues and these images are rich in texture allowing the analysis of image in terms of these features. The texture of an image can be defined as a constant repetition of an element or pattern on the surface of an image which represents its structure [18, 19]. Texture analysis focuses on finding a specific way of representing the hidden characteristics of textures and express them in a simplified and unique form. Grey level co-occurrence matrices (GLCM) of MRI ADC images can be identified as a rich source of statistical texture features which can be utilized in training robust machine learning (ML) models, which is a powerful method that is commonly utilize to identify the unique patterns of the distribution of texture features within an image [20–22].

GLCM texture feature extraction can be defined as a statistical method that reveals specific properties about the spatial distribution of gray levels in image texture considering the spatial relationship of pixels [23]. Here measures the relation of grey intensities between two adjacent pixels [reference pixel (*i*), neighbor pixel (*j*)] of an image at a time to have information about variation in intensity at a pixel of interest. The GLCM matrices are computed using two parameters such as the relative distance between the pixel pair and the relative orientation (angular relationship) of the pixel pair. Most frequently, the orientation quantified as 0$^{\circ }$, 45$^{\circ }$, 90$^{\circ }$ and 135$^{\circ }$ angles and the average of the resultant values for all four directions used to extract the features [24, 25].

### Higher order moments

Higher order moments can be identified as functions that use high power of a sample (higher than second-order statistics), that is opposed to the conventional first- or second-order statistics (lower order statistics). The higher order statistic provides powerful tools in identifying problems in non linear systems [26]. However, skewness and kurtosis are the examples of third-order and fourth-order statistics, respectively’ [8, 27]. Here, skewness measures the asymmetry around the mean of probability distribution of a real valued random variable and the values for skewness can be zero (0), positive (+), negative (−) or undefined. The kurtosis use to describe the shape of a probability distribution of a real valued random variable and measures the tailedness of it. The kurtosis values for any uni-variate normal distributions remain as 3. However, the distributions with kurtosis values more than 3 are considered as *platykurtic* distributions while considering the distributions with kurtosis values less than 3 as *leptokurtic* distributions [28].

The above mentioned features of MRI brain can be extracted from ADC images of brain neoplasms and used by machine learning techniques to train classifiers.

### Machine learning

**Fig. 1:**
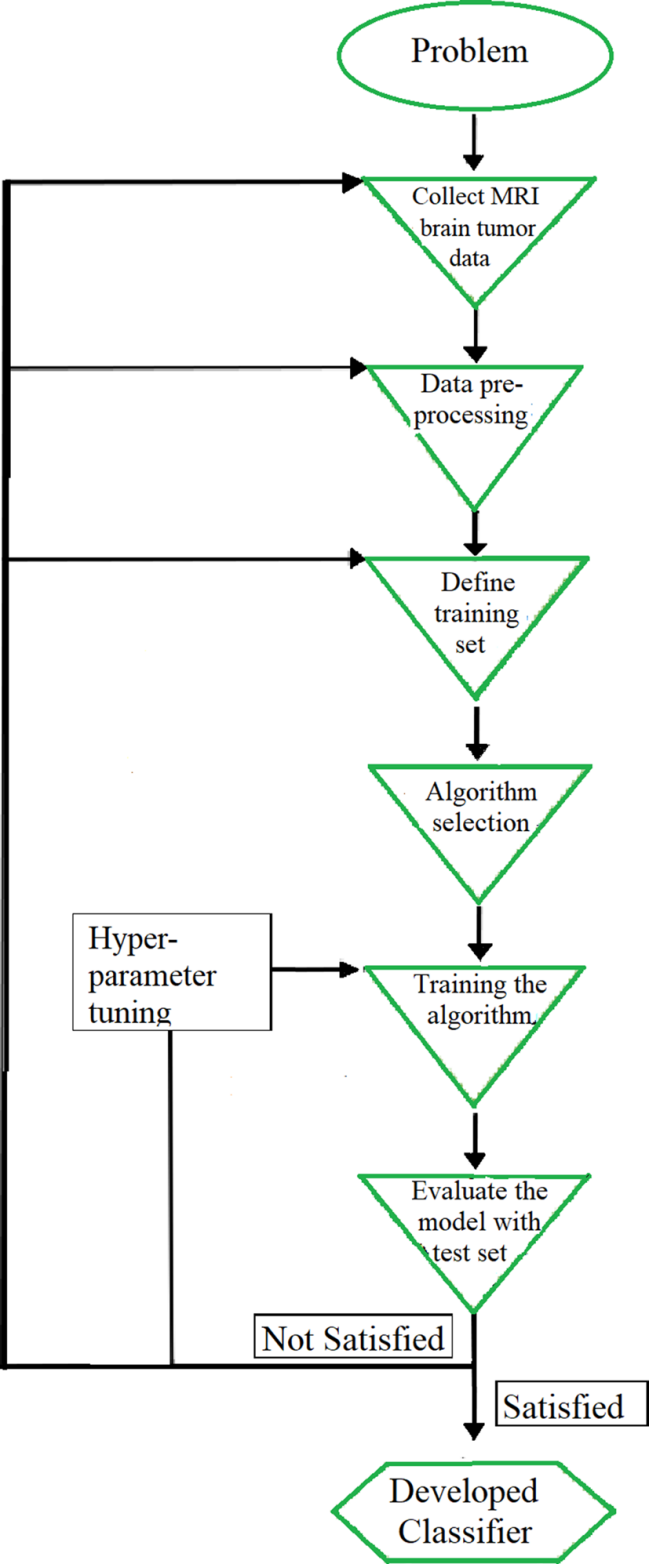
Supervised learning method applying to tumor classification. The flow chart illustrates the steps of building a classification model to differentiate brain neoplasms using supervised learning technique. Here, the problem was identified as a classification problem at the initial stage and then the necessary data was collected as the second step. Data pre-processing was executed as the third step and at the fourth step, the data set was split into training and testing sets. Then a suitable ML algorithm for the collected data was selected as the fifth step of the study flow and then, the selected algorithm was trained with the training data as the sixth step. Finally, the developed algorithm was evaluated with the test data and the hyperparameter of the developed model was tuned to reach the optimum accuracy level of the model

Machine Learning (ML) is a scientific area that allows computers to “learn” from data. The algorithms are used to find out natural patterns in data aiming to aid and/or support decisions and predictions. Considering the goal and the nature of data, ML methods can be further classified as supervised learning, unsupervised learning, semi-supervised learning, reinforcement learning, transduction and learning to learn [29]. Among the above mentioned machine learning techniques, the supervised learning is one of the most common ML paradigms that uses known input and output data to train a model [30–32] to solve classification, regression and forecasting problems [29, 33]. In supervised learning, correct answer to the problem is pre-defined and the ML algorithm identifies the pattern within data correlated with the answer to a particular question. The algorithm make predictions using the observed correlations and the predictions are corrected by the operator. The process iterates until the algorithm achieves highest prediction accuracy. The application workflow of supervised learning method to differentiate brain neoplasms is illustrated in a few basic steps in Fig. 1.

### Machine learning algorithms

Logistic Regression, K-Nearest Neighbors (KNN), Linear Discriminant Analysis, Naïve Bayes, Decision Trees and Random Forest are few of the most common supervised learning algorithms frequently used to solve classification problems.

Logistic regression is a ML algorithm that is designed to solve classification problems by mapping functions from attributes of a data set to its targets. The developed functions are introduced to new examples and predict the probability that the new example belongs to one of the target classes.

K-Nearest Neighbors algorithm is a supervised machine learning algorithm that can be applied on both regression and classification problems. The KNN algorithm assumes that similar data points in a data set exist nearby.

Linear Discriminant Analysis is a ML algorithm that was developed to find a linear combination of features of a data set that separates the data set into two or several classes.

Naïve Bayes is a learning algorithm that is based on the Bayes’ rule to solve classification problems. To apply the Naïve Bayes algorithm, it is crucial to assume that the attributes are conditionally independent in each class. In practice, the above assumption is frequently violated and yet provides competitive classification accuracy [34].

Decision tree is a simple algorithm that often applied to solve both classification and regression problems. It represents the decision workflow to identify the class of an instant within the data set. The algorithm creates the most suitable decision tree models for a training data set by placing the name of the class and specific tests that partitions the space of instances on each node in the process of learning simple decision rules.

Random Forest is a meta estimator that generates a cluster of decision trees on various sub-samples of the provided data set aiming at improving the prediction accuracy of the model while controlling over-fitting by averaging. The variables and thresholds that control the number of decision trees create during the learning process, the maximum number of features considered in splitting a node, maximum number of levels included in each decision tree within the algorithm, the minimum number of data points contain in a node prior to split the node, the minimum number of data points allowed to remain in a leaf node, sampling method of the data points (without or with replacement) are optimized as the model returns highest accuracy level avoiding over-fitting.

### Objectives

The main goal of this study is to propose a Machine Learning classification model that can be used to differentiate benign and malignant brain tumors using different types of features: demographics of patients and statistical ones extracted from ADC images of malignant and benign brain tumors. Statistical features include GLCM texture [1, 35, 36] and higher order moments: skewness (third-order statistics) and kurtosis (fourth-order statistics) [8, 21].

### Main contributions

The main contributions of this work are:

- A collection of 1599 MRI image slices, gathered from 195 patients with brain tumors;
- A vector based data set, where each observation describes a slice and the corresponding type of tumor. This vector is composed of 2 demographic attributes (describing the patient) and 14 numerical attributes (describing the texture of the tumor region), plus the tumor classified as malignant or benign;
- The ANOVA *f*-test analysis of the statistical measures extracted from the MRI-ADC images;
- The proposal of a Machine Learning model that, given a patient tumor, described by a set of MRI image slices, predicts if the tumor is malignant or benign; this model has an estimated precision of 85% and recall of 92% for malignant tumors.
- The developed machine learning model is optimized/tuned as it returns best precision and recall scores.

## Previous studies

In literature, there are several studies can be found that utilized the GLCM statistical texture features of medical images to differentiate benign and malignant tumor types with the assistance of ML algorithms. The study conducted by Xian et al. [37] in year 2010 found the possibilities for utilization of GLCM texture features of ultrasound images to identify malignant and benign liver images and they could predict the tumor type with 97% using fuzzy support vector machine (FSVM) learning method. According to the study conducted by Mohanty et al. [38] able to develop a computer-aided classification model with 94.9% accuracy to differentiate benign and malignant breast carcinomas by analyzing GLCM texture features extracted from digital mammograms of breast carcinomas. Vaidehi et al. [39] in the year 2015 developed an automated breast mass characterization system using the GLCM texture features extracted from mammograms of breast tumors, and the model developed using sparse representation classifiers was able to predict malignant and benign breast tumors with an accuracy of 93.75%. According to the study [40], the researchers were able to utilized two automated methods; artificial neural network (ANN) and cellular neural network (CNN), to differentiate benign and malignant breast carcinomas. At the first stage, CNN was utilized to select the appropriate features among the, intensity, and shape features of the mammograms and the classification problem was addressed by applying ANN. As a result the study was able to make predictions with 96.47% accuracy at high sensitivity (96.87%) and specificity (95.94%).

In addition, several recently conducted studies utilized the GLCM statistical texture features of MRI brain tumors to distinguish brain tumor types, including benign and malignant brain tumors. In year 2014, Preethi et al. [41] implemented the probabilistic neural network with radial basis function (PNN–RBF) to analyze GLCM texture features of brain images of MRI and classify the brain images into healthy, benign, and malignant brain categories. In addition, in year 2016, Kumar et al. [24] developed a computer assisted diagnostic method to demarcate benign, malignant and healthy brain tissues using GLCM texture features of MRI brain images. Here they have observed the differences of values for each type brain pathology/healthy.

Sharma et al. [30] in the year 2014 proposed an automated method to detect brain tumors in MRI images. The researchers extracted the GLCM texture features and fed the feature values into two learning classification algorithms; Multi-Layer Perceptron (MLP) and Naive Bayes for classification. However, the developed classification model using MPL has classified the normal and abnormal brain images with an accuracy of 98.6% and, the Naive Bayes classification model classified the normal and abnormal brain images with an accuracy of 97.6%.

Jain [42] developed a classification model using Artificial Neural Network (ANN) that classified the types of MRI images of astrocytomas. The extracted statistical texture features of GLCM were fed into algorithm. Here the back propagation algorithm was utilized in the learning process while using the tan-sigmoid (tansig) function for the hidden layer and logsigmoid (logsig) function for the output layer.

Byale [43] in 2018, introduced an automated system to classify the MRI images of brain tumors in to benign and malignant categories. They utilized the Gaussian Mixture Model (GMM) to find the region of interest (ROI) and the GLCM texture features of the selected ROIs were extracted. The Neural Networks (NN) was trained using the extracted texture features and the developed model was able to classify tumors with the accuracy of 93.33%.

Compared to the literature, the authors of this study have utilized a novel technique to develop an automated method using ML to differentiate benign and malignant brain tumors by extracting GLCM texture features from the ADC images of brain tumors.

## Results

According to Table 1 skewness (3.3444) and GLCM Homogeneity (3.4572) reported the minimum scores at the ANOVA *f*-test feature selection experiment while the feature “patient gender” reporting the highest (73.7926) score (see Fig.  4).

**Table 1:** ANOVA *f*-test feature selection

Feature	ANOVA *f*-test score (mean value)
Mean pixel value of ADC	32.3343
Skewness	3.3444
Kurtosis	9.6250
GLCM mean 1	32.6372
GLCM mean 2	29.1327
GLCM Var 1	14.0761
GLCM Var 2	27.5219
GLCM energy	33.9675
GLCM entropy	4.989
GLCM contrast	47.9462
GLCM homogeneity	3.4572
GLCM correlation	48.6392
GLCM prominence	15.4134
GLCM shade	17.1677
Patient age	9.4337
Patient gender	73.7926

The table visualize the performance of each feature at the ANOVA *f*-test Feature Selection process. The data set went through the ANOVA *f*-test Feature Selection process for 5 times and the mean values were calculated. There were slight differences of values received at each time due to stochastic nature of the algorithm, or differences in numerical precision or evaluation procedure

As a result of ten-fold cross-validation experiment for the training and testing data sets, the Random Forest Classifier expressed the highest accuracy level (84.36%) while Logistic Regression, Linear Discriminant Analysis, k-Nearest Neighbors Classifier, GaussianNB and SVC obtained 75.33%, 74.89%, 82.84%, 80.07%, 74.89%, and 81.50% accuracy levels, respectively (see Table  2). Hence, the study was continued through the Random Forest Classifier to build most accurate ML model to differentiate malignant and benign brain tumor MRI–ADC images.

**Table 2:** Results of the cross validation experiment

Algorithm	Mean accuracy	Accuracy as percentage (%)	Standard deviation (SD)
Logistic regression	0.753378	75.33	0.034451
Linear discriminant analysis	0.748898	74.89	0.036810
k-Nearest neighbors classifier	0.828459	82.84	0.030710
Decision tree classifier	0.800764	80.07	0.045553
GaussianNB	0.748922	74.89	0.052582
SVC	0.815082	81.50	0.043396
Random forest classifier	0.843629	84.36	0.042054

The table visualize the performance of each machine learning algorithm received at the cross-validation experiment over the training data set and the standard deviations for each result

As a result of training the Random Forest Classifier, the generated model acquired the ability to predict the tumor type (malignancy and benign status) with 85% accuracy level. The model performance over the test data is presented in Table 3 and revealing that the ML model was able to identify the malignant tumors with 85% Precision, 92% Recall which corresponds to a 89% F1-score. Moreover, a performance of 85% for Precision and 73% for Recall (which corresponds to a F1-score of 79%) was obtained for the benign tumors.

**Table 3:** Classification report (without optimizing the model) shows a binary classification of the data set with Random Forest Classifier

Tumor type	Precision (%)	Recall (%)	F1-score (%)	Support
Malignant	85	92	89	299
Benign	85	73	79	181
Accuracy			85	480
Macro average	85	83	84	480
Weighted average	85	85	85	480

The best hyper-parameters for maximum Precision were $max\_depth=70$, $max\_features=10$, $min\_samples\_split=2$ and $n\_estimators=500$, and the ML model predicted 229 malignant and 117 benign tumors correctly with 34 false negatives and 20 false positives from the test data (see Table 4). The best parameters which returned the maximum recall score was $max\_depth$: 30, $max\_features$: 10, $min\_samples\_split$: 2, $n\_estimators$: 300 (see Table  5) and 231 malignant tumors, 119 benign tumors were accurately predicted while expressing 32 fails negatives and 18 fails positives (see Table 6).

**Table 4:** Classification report: performance of Random Forest after hyperparameter optimization to have best precision score

Tumor type	Precision (%)	Recall (%)	F1-score (%)	Support
Malignant	89	94	92	299
Benign	90	81	85	181
Accuracy			89	480
Macro average	89	87	88	480
Weighted average	89	89	89	480

**Table 5:** Optimum level of hyper parameters for maximum precision score and the maximum Recall score for the selected features, where n estimators is the number of trees in random forest, min sample split is the minimum number of samples required to split a node and max depth is the maximum number of levels in tree

Hyper parameter	Best condition for Precision	Best condition for Recall
*n* estimator	500	300
Min sample split	2	2
Max features	10	10
Max depth	70	30

**Table 6:** Classification report: performance of Random Forest after hyperparameter optimization to have best recall score

Tumor type	Precision (%)	Recall (%)	F1-score (%)	Support
Malignant	91	93	92	299
Benign	88	85	86	181
Accuracy			90	480
Macro average	87	85	86	480
Weighted average	87	87	87	480

**Fig. 2:**
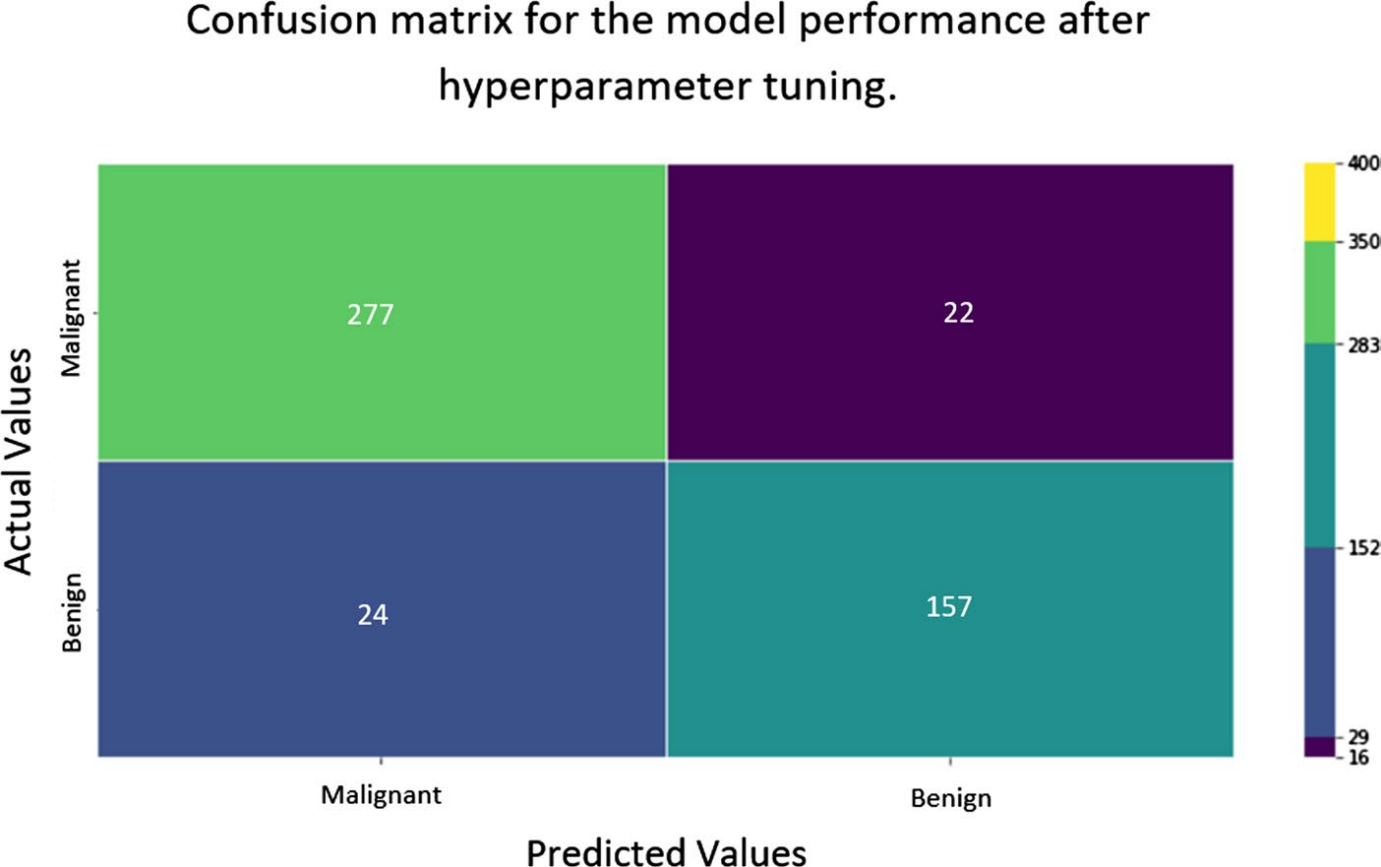
Final confusion matrix. The confusion matrix express the performance of the optimized benign malignant brain tumor brain tumor classification model over the test set

Finally the decision threshold was adjusted to 0.45 with the assistance of the information provided by the precision and recall curve (see Fig. 5). As a result, the accuracy score of the optimized ML classification model increased up to 90.41% while the precision, recall and f1 score for predicting malignant tumors maintaining at 92.02%, 92.64% and 92.33%, respectively. In addition, for the benign tumor prediction, the precision, recall and f1 scores reported as 87.71%, 86.74% and 87.22% (see Fig. 2).

## Discussion

The excellent soft tissue differentiation ability of MRI allows to visualize the exact location of the tumor, and aids to therapeutic, diagnosis and evaluation process of human brain tumors [30, 44, 45]. Moreover, the modern MRI techniques used in clinical setup such as DWI, DTI and DSCI utilize to assist in tumor characterization and treatment process [46]. This study focused on developing an automated method using currently available MRI technique (DWI) to differentiate malignant and benign brain tumors. However, developing an accurate, automated and noninvasive method to differentiate benign and malignant brain tumors leads to increase the accuracy of the diagnosis process in terms of sensitivity and the specificity. Since such a tool has the ability to assist the clinician in decision-making at the brain tumor diagnosis process, it can be applied as an intermediate step in between tumor imaging and the brain biopsies which allows the clinician to decide the necessity of requesting a biopsy for further investigation.

To achieve these goals, a ML model was developed using extracted features from DWI images and the demographics of patients. The mean pixel value, skewness, kurtosis, GLCM features (mean, variance, energy, entropy, contrast, homogeneity, correlation, prominence and shade values) and demographic features (age and gender) were extracted using BLeDIA, an home made software which was specifically designed for this study. We hypothesized that the above mentioned features correlate with malignant and benign brain tumors in complex and non-linear ways. Therefore, the combination of all features except skewness and GLCM homogeneity were used to develop a machine learning model able to distinguish the malignant and benign status of brain tumors. As a byproduct of ANOVA *f*-test feature selection process, we have observed that the feature “patient gender” showed the highest ANOVA *f*-test score (73.7926) (see Table 1). Therefore, it is possible to assume that the patient’s gender has high impact on predicting benign and malignant brain tumors. Such information can be studied as an extension of this study.

The Selected normalized features were used on several classification algorithms to find out the best fit algorithm for the data set. According to performance obtained (presented in Table 2) cross validation test, the Random Forest Classifier showed the highest score being then selected to develop a ML model.

The Random Forest Classifier could predict the malignant and benign brain tumors with 85% accuracy level (see Table  3). However, the accurate interpretation is not straightforward due to the numeric nature of the extracted features. Therefore, hyper parameter tuning and the decision threshold adjustment was utilized to increase the overall accuracy level of the ML model in terms of sensitivity and specificity. As visualized in Table 5 the optimized Hyper parameter values for precision and the recall was measured. However, according to precision recall curve (see Fig. 5) and the ROC curve (see Fig. 6) the decision threshold value was adjusted as the ML model returns the optimum precision and recall values. As result of tuning the ML model in two steps, finally the ML model able to predict the malignant and benign brain tumors with 90.41% accuracy level with high recall score (92.64%) for malignant tumor identification which indicates that there is less probability to not detect malignant tumors (see Fig. 2).

## Conclusions

The study concludes that mean ADC, kurtosis of ADC and the GLCM features of ADC (mean, variance, energy, entropy, contrast, correlation, prominence and shade) and demographics features can be used as potential bio-markers to identify and differentiate benign and malignant brain tumors.

Given the findings just presented, one can say that this study reveals that there is a great potential on using the developed ML mode in clinical practices to differentiate benign and malignant brain tumors. The results of this study encourage to develop an advanced ML model to predict WHO grading of brain tumors and specifically identifying brain tumors.

## Methods

This prospective study was designed to address the above mentioned objectives of the study and hypothesized that there is a correlation between the extracted features and the benign and malignant status of the tumors. According to the nature of the features extracted, the study plan was designed and Fig. 1 summarises the supervised learning process which was used to develop a robust automated technique to discriminate malignant from benign brain tumors.

### Data acquisition and preparation

This study includes 1599 MRI brain image slices from 195 patients of both sexes (53.41% male and 46.59% female) and all the subjects were within the 12–80 year age range with an average of 45.51 years. The MRI Digital Imaging and Communications in Medicine (DICOM) data of each subject was acquired after confirming the pathological condition by referring both radiological and histopathological reports of each patient. All patients data was obtained from the Departments of Radiology and Histopathology, National Hospital of Sri Lanka (NHSL) and Anuradhapura Teaching Hospital, Sri Lanka, followed by the informed consent of the patients and ethical clearance certificate from the institutional ethical review committee of NHSL and the Faculty of Medicine, University of Peradeniya.

From the initial set of 1896 ADC image slides, 297 were removed according to the exclusion criteria such as lack of information, corrupted MRI images, and the selected tumor not within the considered area (Brain). Therefore, the study was conducted with the reaming 1599 image slices which was consisted with 62.22% malignant tumours and 37.77% benign brain tumor slices (see Table 7).

**Table 7:** Tumor types and percentages belonging to each benign and malignant categories

Category	WHO grading	Tumor type	Image slices	Percentage (%)
Benign	WHO I	Meningioma	262	43.38
		Schwannoma	135	22.35
		Pilocytic astrocytoma	13	2.15
		Hemangioblastoma	16	2.65
		Craneopharyngioma	11	1.82
		Dermoid cyst	13	2.15
	WHO II	Low grade gliomas	112	18.54
		Meningioma	21	3.48
		Astrocytoma	10	1.67
		Ependymoma	7	1.16
		Frontal cavernoma	4	0.66
Malignant	WHO III	High grade gliomas	147	14.77
		Anaplastic astrocytomas	22	2.21
		Anaplastic meningioma	11	1.10
		Anaplastic oligodendro glioma	29	2.91
		Central astrocytomas	65	6.53
	WHO IV	Glioblastomas	442	44.42
		Medulloblastoma	109	10.95
		Metasasis	170	17.08

According to the radiological and histopathological reports, there were 995 malignant brain image slices, including WHO (World Health Organization) Grade IV tumors; 442 glioblastomas, 109 medulloblastoma, 170 metasasis/residual malignancies, and WHO Grade III tumors; 147 high grade gliomas, 22 anaplastic astrocytomas, 11 anaplastic meningioma, 29 anaplastic oligodendro glioma, 65 central astrocytomas within the population. Also there were 604 benign brain tumors slices with WHO Grade I; 13 pilocytic astrocytoma, 262 meningioma, 135 shwannoma, 16 hemangioblastoma, 11 craneopharyngioma, 13 Dermoid cysts, and WHO Grade II; 10 astrocytoma, 21 meningiomas, 112 low grade gliomas, 7 ependymomas, 4 frontal cavernoma

All scans in this study were performed with a 3T Siemens Skyra MR system using head coil and utilized the EPI (Echo Planner Imaging) sequence to acquire axial DW MRI data in both $b = 0$ and $b = 1000$ diffusion sensitization levels with a $flip\_angle=90^{\circ }$, $\text{TE}=68\,{\text{ms}}$ and $\text{TR}=4300\,{\text{ms}}$ (being TE the time of echo and TR the time of repetition), $\text{FOV}=219\,\text{mm}\times 219\,{\text{mm}}$, $matrix\_size=124\times 124$ and $slice\_thickness=1\,{\text{mm}}$.

### ADC image generation and ROI selection

The ADC images were generated by merging two different DW images with different diffusion sensitization levels ($b=0$ and $b=1000$) according to Eq. 1, where *i* is the image number, $S_i$ the $i{\text{th}}$ image (image acquired with a diffusion pulse of *i*), $S_0$ the first image (image acquired without any diffusion pulses), *n* the number of images and $b_i$ the diffusion gradient value. A homemade computer program was utilized to achieve all the image processing goals, such as image selection, visualization, ADC image generation, ROI selection and feature extraction. All the ROIs were selected manually under the supervision of consultant radiologists:

1 $${\text{ADC}} ={\sum _{i=1}^{n}}\ \dfrac{\ln \dfrac{S_i}{S_0}}{b_i}$$

**Fig. 3:**
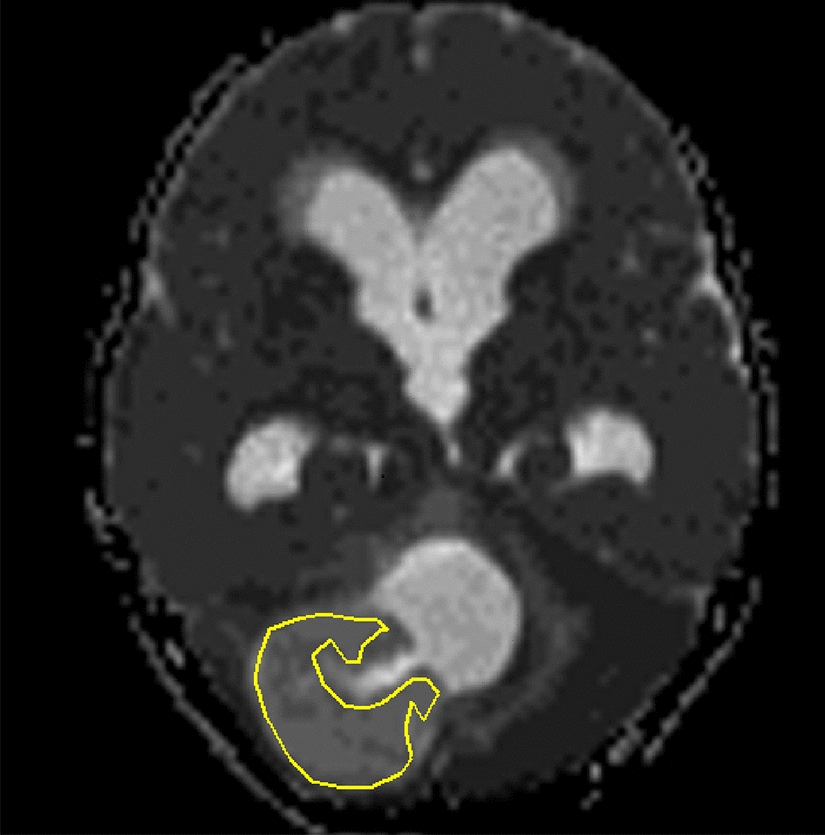
MRI ADC brain image of a 14-year-old female patient diagnosed with pilocytic astrocytoma which was radiologically and histo-pathologically identified as a benign tumor. The tumor area is surrounded by the ROI. The texture features were extracted form the selected area

The tumor area of each 2D ADC image slice was selected by drawing a 3D region of interest (ROI) encompassing the tumor (see Fig. 3) and extracted the pixel values within the selected area.

### Feature extraction

We have evaluated the mean, higher order moments skewness ($n=3$) and kurtosis ($n=4$) and GLCM based statistical texture features of MRI–ADC brain tumors and the patients demographics. The mean pixel value and the higher order moment values were calculated within the ROI using Eqs. 2 and 3, respectively. Here, $P_i$ represents the signal intensity in $i{\text{th}}$ pixel and *N* is the total number of pixels within the ROI, *P* is the mean of the pixel values and $f(P_i)$ the probability of the signal intensity of pixel:

2 $$\text{Mean} = \dfrac{\sum _{i=1}P_i}{N}$$

3 $$n{\text{th}}\; {\text{moment}}= \sum _i (P_i - P)^n f(P_i)$$

MATLAB 2019 Simulink software was used in all the image processing steps and Python 3.7 in all the feature extraction and analysis processes [8]. The GLCM matrices of each 2D parametric map of ADC brain tumor were derived according to Eq. 14 (see Appendix). The statistical texture features of GLCM (mean, variance, energy, entropy, contrast, homogeneity, correlation, prominence and shade values) were extracted from the generated GLCM matrices. Moreover, the GLCM features were extracted according to Eqs. 5 to 13 (see Appendix) [24, 25, 47]:

4 $$M_{f,\delta }(k,l) = \sum _{x,y=1}^n {\left\{ \begin{array}{ll} 1 \text { if } f(x,y)=k \text { and } f(x+\delta _{x,y}+\delta _{y}) = y\\ 0 \text { otherwise} \end{array}\right. }$$

Here the *f* consider as 2D parametric ADC map, $M_{f,\delta } (k,l)$ is the co-occurrence matrix which represents the joint probability occurrence of pixel pairs with grey level value *k* and *l* for, $\delta = (\delta _x,\delta _y)$ specific spatial offset between the pixel pair. *n* is the bar of grey levels in 2D parametric ADC map of brain tumor.

However, lower and higher order moments (see Eq. 3); mean pixel value (*n* = 1), skewness (*n* = 3), kurtosis (*n* = 4) and texture features of GLCM such as mean, variance, energy, entropy, contrast, homogeneity, correlation, prominence and shade values were studied in this pattern recognition process; The GLCM features were extracted according to Eqs.  5,  6, 7, 8, 9, 10, 11, 12 and  13 respectively [24, 25, 47]. Here $P_{i,j}$ be the co-occurrence matrix, *N* be the number of grey levels in the image, $\mu$ be the mean of $P_{i,j}$, $\mu _{i}$ be the mean of row *i*, $\mu _{j}$ be the mean value of column *j*, $\sigma _{i}$ be the standard deviation of row *i* and $\sigma _{j}$ be the standard deviation of column *j*. The extracted feature values were stored in a CSV file for data preparation and further analysis.

#### GLCM mean

Left-sided equation calculates the mean based on the reference pixel $(\mu _i)$ while right-sided equation calculates the mean using neighbouring pixels $(\mu _j)$:

5 $$\begin{aligned} \mu _{i}= \sum _{i,j=0}^{N-1}i\left( P_{i,j} \right) \mu _{j}= \sum _{i,j=0}^{N-1}j\left( P_{i,j} \right) \end{aligned}$$

#### GLCM variance

Left-sided equation calculates the dispersion of the reference pixel values $(\sigma _i^2)$ around $(\mu _i)$ and the right-sided equation calculates the dispersion of the neighbour pixel values $(\sigma _j^2)$ around $(\mu _j)$:

6 $$\begin{aligned} \sigma _{i}^{2}=\sum _{i,j=0}^{N-1}P_{i,j}\left( i-\mu _{i} \right) ^{2} \sigma _{j}^{2}=\sum _{i,j=0}^{N-1}P_{i,j}\left( j-\mu _{j} \right) ^{2} \end{aligned}$$

#### GLCM energy (En)

Energy expresses the uniformity of the texture (within a scale between 0 and 1) by measuring the sum of squared elements in the GLCM. GLCM energy value is 1 when the texture is uniform:

7 $$\text{En} =\sum _{i,j=0}^{N-1}P_{i,j}^{2}$$

#### Entropy (Etr)

Entropy describes the degree of disorder among pixels within the matrix, which is approximately inversely correlated with uniformity. The Larger the number of grey levels within the image express larger entropy values:

8 $$\text{Etr} = \sum _{i,j=0}^{N-1}P_{i,j}\left( -\ln P_{i,j} \right)$$

#### GLCM contrast (Con)

GLCM Contrast expresses the amount of local gray level variation in an image. Presence of edges, noise, or wrinkled textures within an image returns high contrast values:

9 $$\text{Con}=\sum _{i,j=0}^{N-1}P_{i,j}\left( i-j \right) ^2$$

#### Homogeneity (Hom)

Homogeneity expresses the smoothness of the distribution of gray levels within an image, which is approximately, inversely correlated with contrast:

10 $$\text{Hom}=\sum _{i,j=0}^{N-1}\frac{P_{i,j}}{1+\left( i-j \right) ^{2}}$$

#### Correlation (Cor)

Correlation expresses the amount of linear dependency of gray levels among two neighbouring pixels within the matrix. Texture with high GLCM correlation has high predictability of pixel relationships:

11 $$\text{Cor}=\sum _{i,j=0}^{N-1}P_{i,j}\left[ \frac{\left( i-\mu _{i} \right) \left( j-\mu _{j} \right) }{\sqrt{\left( \sigma _{i}^{2} \right) \left( \sigma _{j}^{2} \right) }} \right]$$

#### Cluster shade (Shd)

Cluster Shade evaluates the tendency of clustering of the pixels by measuring the skewness of pixel values within the matrix:

12 $$\text{Shd} = \sum _{i,j=0}^{N-1}\left\{ i+j-\mu _{i}-\mu _{j} \right\} ^{3}P_{i,j}$$

#### Cluster prominence (Prom)

Cluster Prominence measures local intensity variation of pixels and the asymmetry of an image. The high prominence value indicates less symmetry of an image, while image with less cluster prominence value shows peak in GLCM matrix around the mean:

13 $$\text{Prom} = \sum _{i,j=0}^{N-1}\left\{ i+j-\mu _{i}-\mu _{j} \right\} ^{4}P_{i,j}$$

### Feature selection and model training

The full set of image slices was split into train and test subsets (70% and 30% of data, respectively) in a stratified way, resulting in sets of 1119 image slices for training and 480 for testing.

The extracted feature values were normalized utilizing Python 3.7 along with scikit-learn library to have zero mean and unit variance (see Eq.  14, where $X_{n}$ is the feature normalized value, *X* is the feature value and $X_{\min }$ and $X_{\max }$ are the minimum and the maximum values for the particular feature):

14 $$X_{n}= \frac{X-X_{\min }}{X_{\max }-X_{\min }}$$

**Fig. 4:**
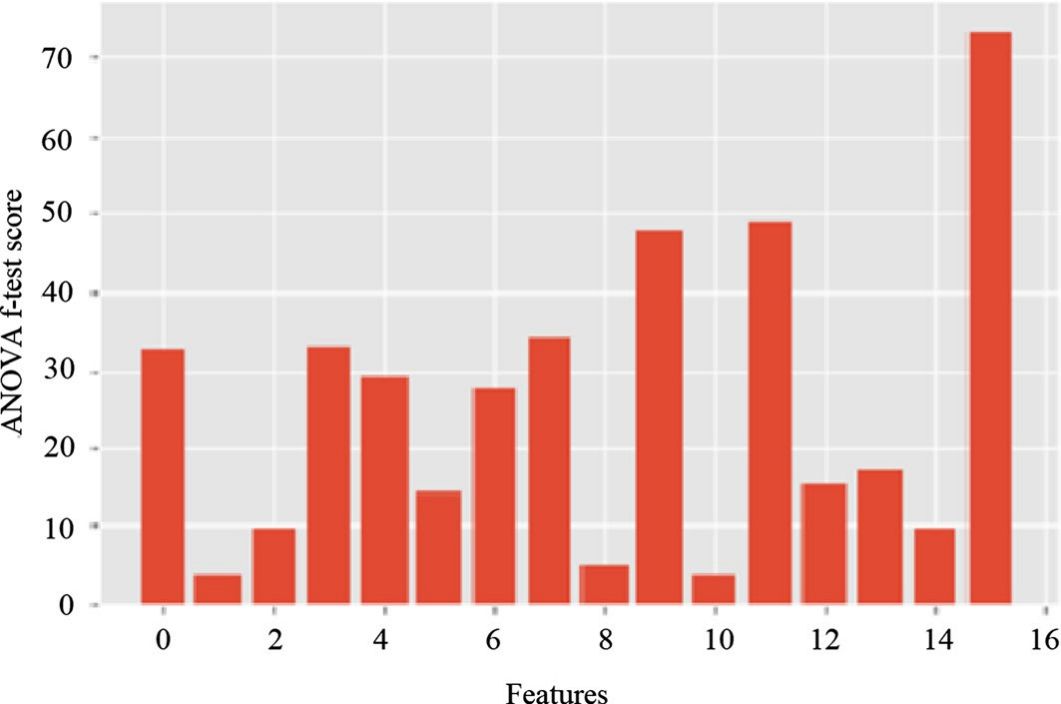
ANOVA *f*-test results chart. ANOVA *f*-test score for attributes 0 to 15 are illustrated in the graph; mean pixel value of ADC 32.3343, Skewness 3.3444 Kurtosis 9.6250, GLCM Mean1 32.6372, GLCM mean2 29.1327, GLCM variance1 14.0761, GLCM variance2 27.5219 GLCM energy, GLCM Homogeneity 3.4572, 33.9675, GLCM Entropy 4.989, GLCM contrast 47.9462, GLCM Correlation 48.6392, GLCM prominence 15.4134, GLCM Shade 17.1677, Patient Age 9.4337 and Patient Gender 73.7926

A subset of the normalized features most relevant to the target variable (malignant or benign) was selected using the ANOVA (Analysis of Variance) *f*-test technique. To reduce the effect of the stochastic nature of the algorithm and differences of numerical precision, the process was repeated 5 times and the mean score for each attribute was calculated (see Table 2). Figure 4 presents them graphically [48].

A tenfold cross-validation method was used to figure out the most promising algorithm at discriminating malignant and benign brain tumors. The following algorithms were tested using the default parameters in all of them: Logistic Regression, Linear Discriminant Analysis, K-Nearest Neighbor, Decision Tree Classifier, Gaussian Naive Bayes (GaussianNB), Support Vector Classifier (SVC) and Random Forest. The results are presented in Table 3.

### Parameter tuning and prediction

Then the selected normalized features were fed into the Random Forest Classifier to develop a tumor classification model and the performance was evaluated for different parameters of the algorithm. The accuracy, Precision, Recall and F1 measures obtained with the set of parameters that produced the best model is presented in Table 4.

To search for the best set of parameters, a random grid search was performed aiming to increase the tumor prediction accuracy and a decision threshold adjustment was done to optimize the sensitivity and specificity of the classification model [49]. The considered tunable hyperparameters of the algorithm were: $min\_samples\_split$, $n\_estimators$, $max\_depth$ and $max\_features$ and each hyper parameter was tested within a pre-defined ranges of values ($min\_samples\_split$: [2, 5, 10], $n\_estimators$: from 200 to 1000 (with step of 10), $max\_depth$: 10 to 100 (with step of 10), and $max\_features$: [3, 5, 10, 20]). Here the optimum values for each hyperparameters that maximize the precision and recall of the developed classification model were measured separately (see Table 7).

**Fig. 5:**
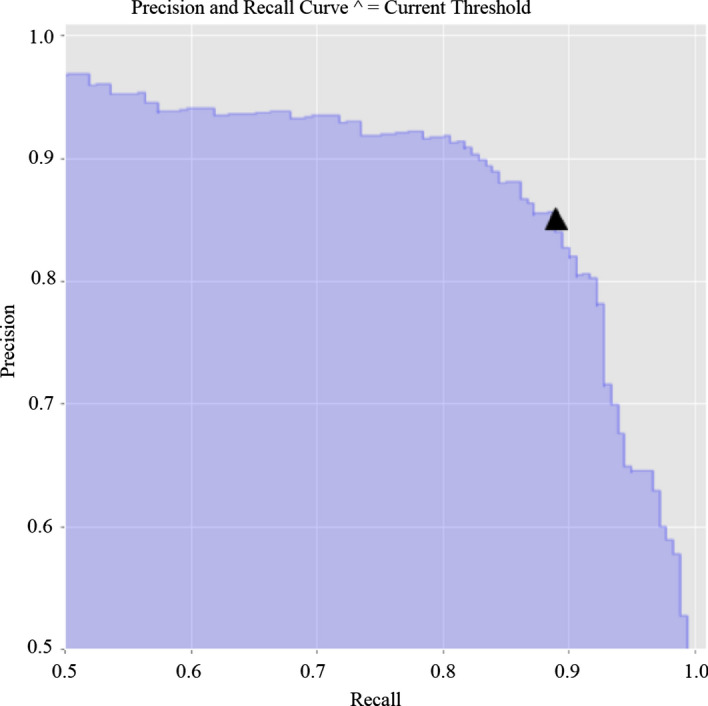
Precision–recall curve; visualize the sensitivity–specificity trade-off in the classifier the information provided by the curve used to set the decision threshold of the model to maximize the sensitivity and specificity

**Fig. 6:**
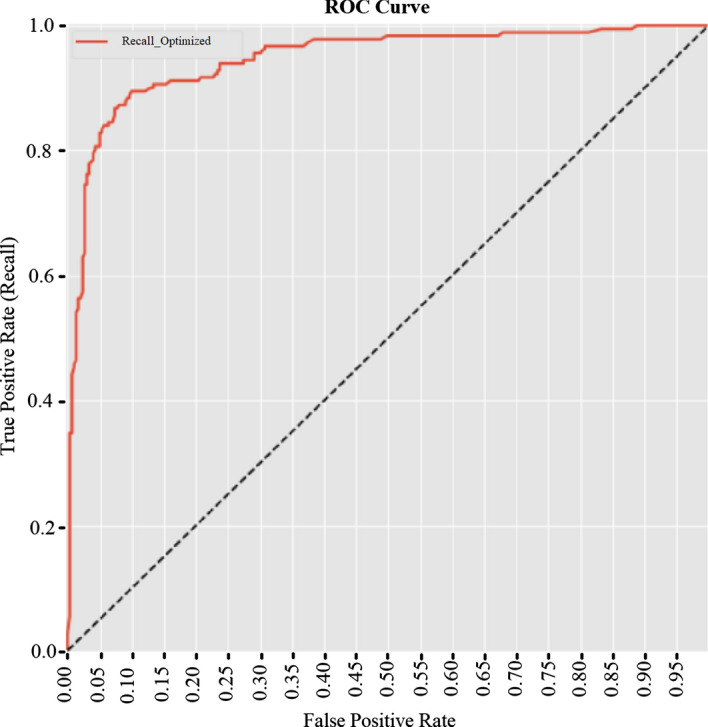
Receiver operating characteristic curve (ROC Curve). The curve illustrates the behaviour of the false positive rate (*x*-axis) and true positive rate (*y*-axis) for a series of different decision threshold values in between 1.00 and 0.00. The smaller values of the *X*-axis represent the lower false positive rate, and the higher true negative rate. In addition, the larger values of *Y*-axis represent the lower false negative rates and higher true positive rates

The decision threshold (the operating point) of the developed ML model was adjusted to improve either sensitivity or specificity of the model. It was adjusted with the guidance of the precision–recall curve shown in Fig. 5 and the receiver operating characteristic (ROC) curve utilized to estimate the performance of the developed classification model (see Fig. 6). In addition, performance of the tuned classification model was assessed by observing accuracy score, precision, recall and F1 scores over the test set (Eqs. 16, 17, 18):

15 $$\text{Accuracy} = \frac{\text{TP} + \text{TN}}{\text{TP} + \text{TN} + \text{FP} + \text{FN}}$$

where TP, TN, FP, and FN indicate True Positive, True Negative, False Positives and False Negatives, respectively. The accuracy express the proportion of all correct prediction from the total number of predictions made by the machine learning model:

16 $$\text{Precision} = \frac{\text{TP}}{\text{TP}+\text{FP}}$$

where TP is true positives and FP indicates the false positives. Precision indicates the performance of a machine learning model by measuring the quality of positive predictions:

17 $$\text{Recall} = \frac{\text{TP}}{\text{TP}+\text{FN}}$$

where TP is true positives and the FN indicates false negatives. Recall measures the correctly predicted positive cases out of all the positive individuals:

18 $$F_{1} = 2\cdot \tfrac{\text{Precision} \cdot {\text{Recall}}}{\text{Precision}+ {\text{Recall}}}$$

The harmonic mean of precision and recall is represent by the F$_{1}$ score

The precision and recall curve (see Fig. 5) utilized to visualize sensitivity and specificity trade-off in the classifier. With the assistance of the information from precision and recall curve, the decision threshold was adjusted and set to 0.45 which maximize the sensitivity and specificity of the developed ML model. The ROC curve (see Fig. 6) was implemented visualize the performance of the ML model at all classification thresholds. As a result of adjusting the decision threshold and the hyperparameter tuning process, the prediction accuracy of the developed ML model increased up to a considerable level.

## Data Availability

The data that support the findings of this study are available on request from the corresponding author [M.L Jayatilake]. The data are not publicly available due to them containing information that could compromise research participant privacy, and consent.

## Appendix

See Figs. 5 and 6.

## Abbreviations

MRI: Magnetic resonance imaging
DW: Diffusion weighted
DWI: Diffusion weighted imaging
CNS: Central nervous system
ADC: Apparent diffusion coefficient
GLCM: Grey Level Co-occurrence Matrix
ML: Machine learning
ROC: Receiver operating characteristic curve
